# Effect of Maillard reaction on biochemical properties of peanut 7S globulin (Ara h 1) and its interaction with a human colon cancer cell line (Caco-2)

**DOI:** 10.1007/s00394-013-0494-x

**Published:** 2013-01-20

**Authors:** Małgorzata Teodorowicz, Ewa Fiedorowicz, Henryk Kostyra, Harry Wichers, Elżbieta Kostyra

**Affiliations:** 1 Department of Biochemistry, Faculty of Biology, University of Warmia and Mazury, Oczapowskiego 1A, 10-719 Olsztyn, Poland; 2 Cell Biology and Immunology Group, Wageningen University and Research Centre, De Elst 1, 6708 WD Wageningen, The Netherlands; 3 Department of Immunology and Food Microbiology, Institute of Animal Reproduction and Food Research of the Polish Academy of Sciences, Tuwima 10, 10-747 Olsztyn, Poland; 4 Food Chemistry Group, Wageningen University and Research Centre, P.O. Box 8129, 6700 EV Wageningen, The Netherlands; 5 Food and Biobased Research, Wageningen University and Research Centre, P.O. Box 17, 6700 AA Wageningen, The Netherlands

**Keywords:** Ara h 1, Food allergy, Maillard reaction, Maillard reaction products (MRPs), IL-8, Caco-2 proliferation

## Abstract

**Purpose:**

The purpose of this study was to determine the influence of Maillard reaction (MR, glycation) on biochemical and biological properties of the major peanut allergen Ara h 1.

**Methods:**

Three different time/temperature conditions of treatment were applied (37, 60, and 145 °C). The extent of MR was assessed by SDS-PAGE, loss of free amino groups, fluorescence intensity, content of bound sugar and fructosamine. The Caco-2 model system was applied to study effects of hydrolysed and non-hydrolysed Ara h 1 on proliferation and interleukin-8 (IL-8) secretion from Caco-2 cells.

**Results:**

We demonstrated significant differences in the biochemical properties of Ara h 1 glycated at different time/temperature conditions. Glycation of Ara h 1 at 37 °C was shown to cause least biochemical changes, not limiting pepsin hydrolysis. Loss of free amino groups, increase of fluorescence and brown colour of Ara h 1 glycated at 60 and 145 °C indicated advanced and final stages of MR. Non-treated Ara h 1 inhibited Caco-2 cell proliferation and stimulated IL-8 secretion. This effect was less pronounced for glycated Ara h 1. Incubation of Caco-2 cells with non-hydrolysed Ara h 1, glycated at the temperature of 37 and 60 °C, did not stimulate IL-8 secretion.

**Conclusion:**

Each applied time/temperature-treatment combination caused different biochemical changes of Ara h 1, underlining diversity of formed MRPs. MR, independently of temperature/time conditions, reduced the pro-inflammatory properties of native Ara h 1, reflected in stimulation of IL-8 secretion from intestinal epithelial cells.

## Introduction

The allergy to peanuts (*Arachis hypogea*) is one of the most common in industrialized societies. Research on the prevalence of peanut allergy carried out under the integrated project on food allergy EuroPrevall showed the occurrence of sensitization to peanut in adult population to be 9.3 % in USA and between 4.2 and 0.8 % in European countries [1]. However, the level of sensitization reaches approximately 10 % in the population of 8-year-old children in the UK [2]. Clinical peanut allergy is detectable in 1–2 % of the total population [2] with an increasing prevalence in US [3] and stable tendency in the UK [4].

Peanut 7S globulin (Ara h 1) is one of the most potent peanut allergens. It was reported that 75 % of peanut allergic individuals have serum-specific IgE against this protein [5]. The 63.5 kDa monomers of Ara h 1 form a highly stable homotrimer held together primarily through hydrophobic interactions between residues on α-helical bundles located on the ends of each monomer [6, 7]. Koppelman et al. [8] showed rapid hydrolysis of Ara h 1 by pepsin, which has been confirmed by other authors [9, 10]. However, a trimeric complex of Ara h 1 may protect from digestion of a linear IgE epitopes which are located mainly in the areas of the subunit–subunit contacts [11]. Thus, fragments of Ara h 1 containing several intact IgE-binding epitopes are able to reach the gut mucosa and influence the intestinal homeostasis [11].

The Maillard reaction (MR, glycation), a non-enzymatic reaction between free amino groups of protein (usually the ε-amino group of lysine residues) and carbonyl groups of reducing sugars is one of the most widespread modifications of proteins that occurs during food processing. The mechanism of glycation is complex and involves a cascade of chemical rearrangements which finally leads to formation of numerous Maillard reaction products (MRPs) including components that may impact on human health (acrylamide, heterocyclic amines (HCAs), glycation/lipoxidation end products) [12, 13]. Recent studies showing MR as a crucial factor changing the biological properties of food proteins [14–17] highlighted the importance of this reaction not only in an areas of food chemistry [18] and flavour chemistry [19] but also human pathology [17, 20–23]. In our previous studies, we demonstrated the influence of different temperatures of MR (37, 60 and 145 °C) on biological activity and biochemical characteristic of purified hazelnut 7S globulin, Cor a 11. We showed that the MR influences such parameters as sensitivity of Cor a 11 to proteolysis, binding capacity for human IgE or rabbit IgG and degranulation capacity of basophiles [15]. We also demonstrated that roasting of Ara h 1 at 145 °C modulates its allergenicity by increasing the basophil degranulation capacity [16]. However, the effect of roasting on the biological properties appears to be specific for different proteins. Thus, there is a need for answering the question how the processing influence the biological activity of different proteins what is especially relevant in terms of proteins with allergenic potential [16]. In this approach, extremely important is the role of MR occurring in food systems on the biological properties of food allergens especially with respect to their interaction with gut epithelia. Careful planning and management of the conditions under which MR takes place may provide safe food, free from Maillard reaction products (MRPs) that have been described as harmful to health [13, 20].

Caco-2 cells are widely used as model for human epithelium cells [14, 17, 21, 22, 24, 25] since Hidalgo reported that they resemble the morphology and function of human intestine epithelial cells [26]. The Caco-2 model allows to analyse biological activity of food components (e.g. MRPs) towards human intestine epithelial cells, reflected in cell proliferation, enzymatic activity, apoptosis and cytokine secretion. Characterization of food compounds in terms of promotion or inhibition of cell proliferation provides information about physiological conditions and growth rate of the cells, forming the basis for numerous in vitro assays of a specific cell response, for example interleukin secretion [21, 22, 27–29]. Intestinal epithelial cells may affect the local immune system by their cytokine secretion and thus promote inflammatory response. Interleukin-8 (IL-8) has been identified as a key mediator of epithelial immune responses which influences several factors including activation, growth, differentiation, and migration of neutrophils, basophils and T cells [30, 31]. Therefore, the modulation of expression of IL-8 by intestinal epithelial cells via MRPs may provide new information in the field of immunomodulation by food especially in terms of conditions such as food allergy.

In this study, we created a model system to investigate the biological changes of the major peanut allergen Ara h 1 treated with and without glucose under three different temperate/time conditions. As before [15], we decided to use temperatures which are widely used in food industry as well as in experimental work to mimic the Maillard reaction in food. Heating at 37 °C, corresponding to human body temperature, has only a minor effect on protein structure and therefore contributes to gaining further knowledge on protein glycation in the body [32]. Heating at 60 °C changes the secondary and tertiary structure of proteins [33], creates an effective condition for MR [34] and is also applied during food manufacturing [35], while 145 °C is the temperature of peanut roasting [36]. This approach allowed us to characterise the effect of these time/temperature conditions with respect to their effect on biochemical and molecular properties of Ara h 1 including:

1. Physicochemical properties at different stages of MR,
2. Sensitivity to pepsin proteolysis,
3. Molecular interaction with gut epithelium in a model system using the Caco-2 cell line.

## Materials and methods

### Extraction and purification of 7S globulin (Ara h 1) from peanuts

Fresh peanuts (*Arachis hypogea*) from Argentina were obtained from the company De Graaf, Pelplin, Poland. Peanuts were ground and defatted with n-hexane (2 times for 15 min). Ara h 1 was extracted and purified from defatted peanut flour as described previously by Marsh et al. [37] with the following modifications: after the extraction procedure, solid ammonium sulphate was added to the extract to a final concentration of 70 % saturation, and precipitation was carried out during 1 h in an ice bath. The supernatant was collected by centrifugation at 40,000×*g* for 40 min at 4 °C. The precipitation procedure was repeated to a final concentration of 90 % saturation of ammonium sulphate. After centrifugation (40,000×*g*, 40 min, 4 °C) the pellet was collected, dissolved in buffer (1:60 v/v, 20 mM Tris, 500 mM NaCl, 3 mMNaN3, pH 7.5) and dialysed overnight against the same buffer at 4 °C using 10 kDa MWCO dialysis tubing. Further purification was performed by concanavalin A affinity chromatography (Sigma Aldrich, Germany) according to manufacturer’s instructions, and elution was carried out with 50 mM α-D-methylmannoside (Sigma Aldrich, Germany). Peanut 7S globulin (Ara h 1) containing peak fractions were pooled and subjected to gel permeation chromatography using a Superdex Preparative Grade S200 column (GE Healthcare, Buckinghamshire, UK). Pooled fractions were concentrated and dialyzed against PBS by ultrafiltration using an Amicon-stirred cell equipped with a regenerated cellulose membrane with a 10 kDa MWCO membrane (Millipore). The protein concentration was assessed by the BCA-assay (Pierce, USA), and purity was determined by SDS-PAGE as described below.

### Protein glycation

The protein solution was split into seven batches of 10 mg each and contained 0.01 % sodium azide. To three batches, glucose (Sigma Aldrich, Germany) was added in a ratio of 1:2 (w/w protein:glucose) [15, 17]. All seven samples were placed into glass bottles, frozen at -70 °C and lyophilized (Christ, Alpha 1-4 LSC). Next, the lyophilized protein samples (one with glucose and one without) were heated for 7 days at 37 °C or for 3 days at 60 °C in a laboratory incubator (Wamed, C-30G), or for 20 min at 145 °C in a forced convection chamber for thermal research (Wamed, KBC 65 W). One batch was not treated and regarded as the native, non-modified form. After treatment, the samples were allowed to gradually cool to room temperature. Proteins were slowly dissolved in Milli-Q water, centrifuged (5000×*g*, 15 min, 4 °C) and the supernatant was collected and ultrafiltrated (Millipore, Amicon Ultra-15, 10,000 NMWL) against PBS for several rounds to remove the residual glucose and other small molecular weight products. Protein concentration and changes in solubility were assessed by the BCA-assay (Pierce, USA). The change of colour was assessed visually and by the measurement of absorbance at *λ* = 420 nm. An aliquots were stored at −70 °C. All treatments were performed in duplicate.

### Assessment of biochemical changes

#### SDS-PAGE analysis

Native (non-modified) and modified Ara h 1 were separated by SDS-PAGE under reducing conditions. Protein samples in SDS-sample buffer were boiled and loaded (10 μg/lane) on a 12 % polyacrylamide gel and afterwards stained using colloidal Coomassie (Sigma Aldrich, Germany). A molecular weight marker ranging from 6.5 to 200 kDa (Sigma Aldrich, Germany) was included, and molecular masses were calculated by the use of GelScan 1.10 software.

#### O-Phthaldialdehyde (OPA) assay

The OPA assay was performed as described before [15, 16]. Protein samples (250 μg/ml) were mixed with the OPA reagent [38] in a ratio of 1:10 (v/v). The mixed solutions were incubated for 20 min at RT, and the absorbance was measured at 340 nm against a control containing PBS and the OPA reagent. Unreacted amino groups were estimated from a calibration curve established with l-leucine.

#### Anthrone-sulphuric acid colorimetric microassay

The anthrone-sulphuric acid colorimetric microassay was performed as described previously [15, 16]. The protein solutions (250 μg/ml) were mixed in 96-well plates (Sigma Aldrich, Germany) with the cooled anthrone reagent in a ratio of 1:10 (v/v) [39]. A glucose standard curve was used to calculate the sugar content.

#### Nitrobluetetrazolium (NBT) assay

The reagent was prepared as described before by Iwan et al. [15]. The reagent and sample were mixed in the ratio 19:1 and incubated at 37 °C. Fructosamine (320 μM/L) was used as a standard and absorbance was measured in a microplate reader (Asys UVM 340) at 550 nm after 9 and 10 min of incubation. The results were calculated using the following formula: *fructosamine concentration* [*μmol*] = [*(∆A sample* − *∆A blind sample*)/(*∆A calibrator* − *∆A blind sample*)] × *calibrator concentration.*

#### Determination of fluorescence

One millilitre of each sample at a concentration of 1 mg/ml were taken and diluted, to prevent quenching effects, with an appropriate volume of PBS. The solution was measured at an excitation wavelength of 374 nm and emission wavelength of 451 nm which were previously estimated as characteristic for Ara h 1 samples. The fluorescence was determined with the use of a fluorescence spectrophotometer (PerkinElmer LS 50B, GB) and quartz glass cuvette with light path of 1 cm. The device was handled with the software package PerkinElmer FL Data Manager (FLDM). The fluorescence intensity of protein-bound compounds and free fluorescent compounds in the fluid obtained after ultrafiltration of treated Ara h 1 (10 kDa cut-off membrane, see section Protein Glycation) was estimated.

### Assessment of biological changes

#### Protein hydrolysis

For pepsin hydrolysis, the protein solution was adjusted to pH 2.2 using 1 M HCl. Pepsin (3,200–4,500 units/mg protein, Sigma Aldrich, Germany) was dissolved in simulated gastric fluid (0.15 M NaCl, adjusted to pH 2.0 with HCl) and added to the protein solution in a final enzyme-substrate ratio of 1:10 (w/w). The hydrolysis was performed for 1 h at 37 °C with agitation. The enzyme was inactivated by increasing the pH of the samples to 7–8 with 1 M NaOH, and subsequently, samples were directly cooled in an ice bath. The degree of hydrolysis was determined by measuring the increase in the number of free α-amino groups after the hydrolysis using the OPA method as described before [38].

#### Caco-2 cell culture

The human colon cancer cell line, Caco-2, obtained from the American Type Culture Collection (Rockville, MD) were between passages 40 and 50. For all experiments, cells were cultured in DMEM (Sigma Aldrich, Germany), supplemented with 20 % FCS (Invitrogen, France), 1 % NEAA (Sigma Aldrich) and 1 % of gentamycin (Invitrogen, France) at 37 °C in a humidified atmosphere of 5 % CO_2_ in air. The culture medium was changed every 2–3 days. For both proliferation and IL-8 secretion tests, cells were plated at a density of 2.5 × 10^5^ cells/ml in 96-well plates (Becton–Dickinson) and grown for 7 days.

#### Proliferation test

To measure DNA synthesis, Caco-2 cells were incubated in the presence of BrdU (5′-Bromo-2-deoxyuridine) reagent and non-hydrolysed and hydrolysed Ara h 1, respectively, at concentration of 500 μg/ml during 1.5 h. After labelling, BrdU incorporation into cellular DNA was measured by a colorimetric immunoassay using a commercially available cell proliferation ELISA kit (enzyme-linked immunosorbent assay), according to the manufacturer’s instructions (Roche, France). The percentage of proliferation was calculated as:

$$ ( {\text{Abs}}_{\text{sample}} - {\text{Abs}}_{\text{blank}} )/({\text{Abs}}_{\text{st}} - {\text{Abs}}_{\text{blank}} ) \times 100\,\% $$

where Abs_sample_ is absorbance of Caco-2 cells incubated in the presence of Ara h 1 and BrdU, Abs_blank_ is absorbance of Caco-2 cells incubated in pure medium (without Ara h 1 and BrdU) and Abs_st_ is absorbance of Caco-2 cells incubated in the presence of BrdU.

#### Determination of IL-8 secretion

Post-confluent, Caco-2 cells were washed two times with PBS pre-warmed to 37 °C. To measure IL-8 secretion, cells were incubated in the presence of non-hydrolysed and hydrolysed Ara h 1 at a concentration of 25 μg/ml during 24 h. Incubation time and protein concentration were estimated and optimized in previous studies. Each experimental condition was analysed in quadruplicate. The content of IL-8 in media was measured using a commercially available ELISA (enzyme-linked immunosorbent assay) according to the manufacturer’s instructions (BD Biosciences, OptEIA, Pharmingen, San Diego, CA).

### Statistics

Two independent experiments for two different batches of treated Ara h 1 were performed in triplicate. Data were expressed as mean ± SD. For each batch, an individual assays were performed in triplicates for the biochemical analysis and in duplicate for Caco-2 studies. All measurements were done in triplicate for biochemical tests or in quadruplicate in the case of in vitro studies. Statistical analysis was carried out by GraphPad Prism 4 software. A one-way ANOVA test with Tukey post hoc (*p* < 0.05) was used to evaluate the significance.

## Results

### The influence of thermal processing in the presence and absence of glucose on physicochemical properties of Ara h 1

**Fig. 1:**
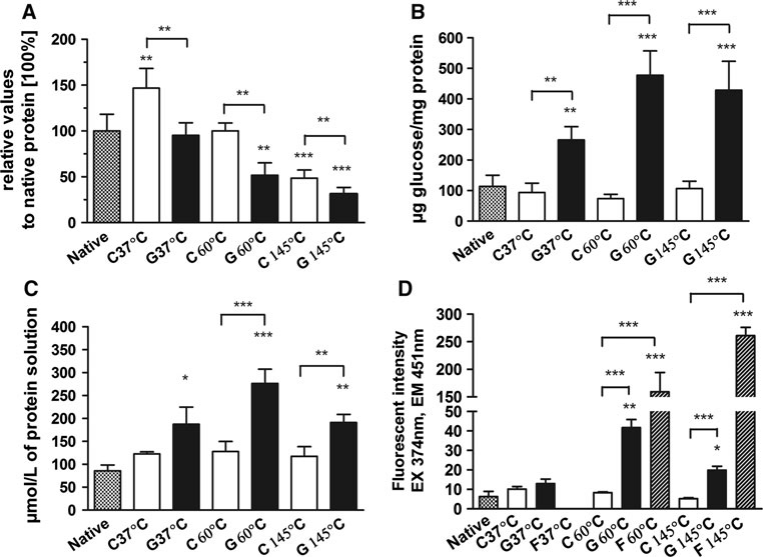
Analysis of the Maillard reaction progress. **a** Percentage of primary amino groups in relation to native Ara h 1 (100 %) determined with OPA method; **b** amount of bound sugar determined by anthrone method; **c** the level of fructosamine determined with NBT assay; **d** the fluorescence intensity of treated Ara h 1 solutions expressed in arbitrary unites (AU). Two independent experiments for two different batches of treated Ara h 1 were performed in triplicate. Data were expressed as mean ± SD. *p < 0.05 *lower part*—statistical differences between treated Ara h 1 and native Ara h 1(N); *upper part*—statistical differences between Ara h 1 treated in the presence of glucose (*G* glycation) and in the absence of glucose (*C* control); F—free fluorescence products obtained due to ultrafiltration of glycated Ara h 1 with the use of 10 kDa cut-off membrane

The extent of Maillard reaction under three different time/temperature combinations was assessed by the use of four methods which taken together give a view of the stage of Maillard reaction and the type of products obtained. For the quantitative determination of free *α*- and ε-amino groups in amino acids, o-phthaldialdehyde in the presence of a thiol component (OPA method) was used. The OPA analysis (Fig. 1a) showed a significant decrease of primary amino groups in the protein heated in the presence of glucose in relation to the control protein heated without sugar in the case of all three time/temperature conditions. However, the treatment at 37 °C in the presence of glucose did not result in loss of free amino groups when compared to native, non-treated Ara h 1. Treatment of protein at both 60 and 145 °C resulted in significant loss of primary amino groups in the relation to native protein. Treatment of Ara h 1 without glucose at 37 and 145 °C also influenced the amount of reactive primary amino groups, most likely due to changes in protein structure. Significant temperature-depended differences in the content of free amino groups were observed in the case of treatments with and without glucose. Treatment of Ara h 1 in the presence of glucose for each time/temperature combination resulted in increased levels of bound sugar compared to both non-treated protein and Ara h 1 heated without glucose (Fig. 1b). Significantly higher amounts of protein-bound glucose were detected in the protein treated at 60 and 145 °C when compared to the one treated at 37 °C. The relative low fluorescence intensity of Ara h 1 glycated at 37 °C (Fig. 1d), a slight decrease of free amino groups content (Fig. 1a), the ratio between fructosamine level and fluorescence intensity (10:1) as well as no colour and solubility changes after the glycation procedure (Table 1) indicate an early and intermediate MR rearrangements. The protein treated at 60 °C in the presence of glucose showed a 4.2-fold higher level of bound sugar compared to the native protein (Fig. 1b) and the highest content of fructosamine (3.2-fold higher, Fig. 1c). The fluorescence absorption of protein-bound compounds was 6 times higher when compared to the native protein and 4 times lower than the intensity of fluorescence of free compounds. The ratio between fructosamine level and protein-bound fluorescent intensity was estimated to be 3:1. Moreover, a light brown colour and a loss of solubility of treated protein were observed (Tab. 1). Those results suggest that under this time/temperature conditions (60 °C, 3 days) MR reached advanced stages. Incubation of Ara h 1 with glucose at 145 °C caused analogous changes in protein properties as the treatment at 60 °C did, characterized by an increased level of bound sugar (3.8-fold higher in relation to the native protein, Fig. 1b), 2.2-fold higher fructosamine level (Fig. 1c) and decreased solubility (Table 1). However, the relation between fructosamine level and protein-bound fluorescence intensity was similar to that observed after treatment at 37 °C. The development of a brown colour correlated with the high intensity of free fluorescent products which was estimated to be 13-fold higher than the intensity of protein-bound fluorescent products and 41.5-fold higher than for the solution of the native protein (Fig. 1d). Temperature/time-dependent differences were observed between Ara h 1 samples treated in the presence of glucose with respect to content of free amino groups, protein-bound sugar and fluorescence intensity. These data show that each of the applied time/temperature treatments promote specific biochemical changes of Ara h 1, reflecting a diversity of formed Maillard reaction products.

**Table 1:** The influence of heat treatment on Ara h 1 solubility and colour changes

	C37 °C	G37 °C	C60 °C	G60 °C	C145 °C	G145 °C
Solubility (%)	99 ± 0.5	97 ± 0.7	87 ± 2.5	59 ± 7.2	68 ± 9.3	8.3 ± 1.4
*p* value	>0.05	>0.05	<0.05	<0.05	<0.05	<0.05
Colour visually	Transparent	Transparent	Transparent	Light brown	Light brown	Brown
Abs. λ = 420 nm	0	0	0	0.1 ± 0.02	0.02 ± 0.00	0.3 ± 0.11

Average percentage of solubility of two heating-treatments performed independently ± SD. *C* control sample, Ara h 1 treated without glucose; *G* glycation, Ara h 1 treated with the presence of glucose

### SDS-PAGE patterns of treated Ara h 1

**Fig. 2:**
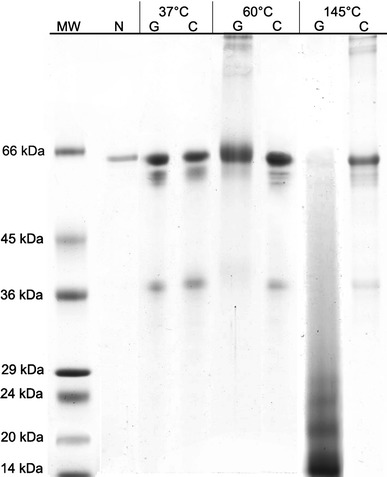
SDS-PAGE patterns of native and glycated Ara h 1. *N* native (non-modified) protein; *C* control sample, Ara h 1 treated without glucose; *G* glycation, Ara h 1 treated in the presence of glucose; *MW* molecular weight marker (kDa)

Quantitative analysis of the SDS-PAGE pattern (Fig. 2, lane 2) and HPLC analysis (data not shown) indicated the purity of the Ara h 1 fraction to be 98.3 %. Figure 2 shows the SDS-PAGE pattern of Ara h 1 before treatment (native, non-modified protein) and after heating in each condition in the presence and absence of glucose. Pure, native Ara h 1 appeared as a single band with a molecular mass of 63,5 kDa (Fig. 2 lane 2) and differed from the patterns of treated Ara h 1 (Fig. 2 lanes 3–8). No differences were observed for the electrophoretic analysis of Ara h 1 heated at 37 °C in the presence or absence of glucose. Regardless of the presence of glucose, approx. 50 % of total protein fraction degraded and was observed as two smaller Ara h 1 fragments with molecular masses of 58 and 36 kDa. Approx. 32 % of Ara h 1 heated at 60 °C in the absence of glucose also degraded into low molecular weight protein fragments (lane 6), while the protein heated in the presence of glucose appeared to be more stable, and 70 % of this fraction (lane 5) was seen with a molecular weight slightly higher than native Ara h 1 (lane 2). Furthermore, electrophoretic analysis of Ara h 1 treated at 60 °C with glucose indicated that 30 % of the total fraction was seen as additional bands with molecular weight ranging from 96 to 110 kDa. Products with a molecular mass higher than 63.5 kDa (estimated for the native form of Ara h 1) were also observed in the pattern of protein treated at 145 °C without glucose, approximately 50 % of total fraction appearing as a 64 kDa band (lane 8). Ara h 1 heated at 145 °C in the presence of glucose appears as a smear at the bottom of the separating gel with a range of molecular masses between 16 and 63.5 kDa where the 63.5 kDa band accounted for only 1.5 % of total fraction.

### The influence of thermal processing in the presence and absence of glucose on enzymatic hydrolysis of Ara h 1

**Fig. 3:**
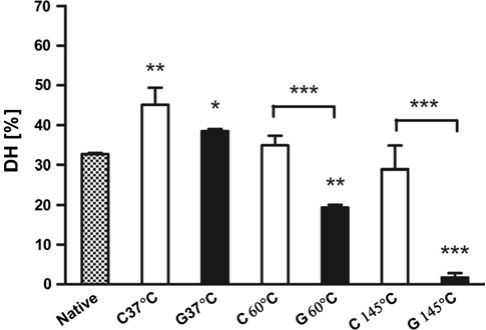
The degree of hydrolysis of Ara h 1 treated without glucose—control (*C*) and with glucose (*G*). Two independent experiments for two different batches of treated Ara h 1 were performed in triplicate. Data were expressed as mean ± SD. *p < 0.05 compared to native Ara h 1 (*lower part*); statistical differences between Ara h 1 treated in the presence and absence of glucose (*upper part*)

The degree of Ara h 1 pepsin hydrolysis (DH) is calculated as the percentage of cleaved peptide bonds (Fig. 3). We observed the temperature/time-dependent differences in susceptibility to pepsin hydrolysis of Ara h 1 treated both with and without glucose. Ara h 1 heated without glucose at 60 or 145 °C did not differ in their degree of hydrolysis. When compared with the native, non-treated protein, the temperature treatment at 37 °C caused 38 % increase of Ara h 1 susceptibility to pepsin hydrolysis, while heating in the presence of glucose increased the degree of hydrolysis by 15 %. The structural changes of Ara h 1 after heating at 60 and 145 °C in the absence of glucose did not change the susceptibility of Ara h 1 to pepsin hydrolysis. Glycation of Ara h 1 at 60 °C resulted in 41 % increase of hydrolysis, while heating at 145 °C in the presence of glucose decreased the degree of hydrolysis to 5 %.

### The influence of glycation of Ara h 1 on human colon cancer cell line Caco-2

#### Proliferation of Caco-2 cells incubated with Ara h 1

**Fig. 4:**
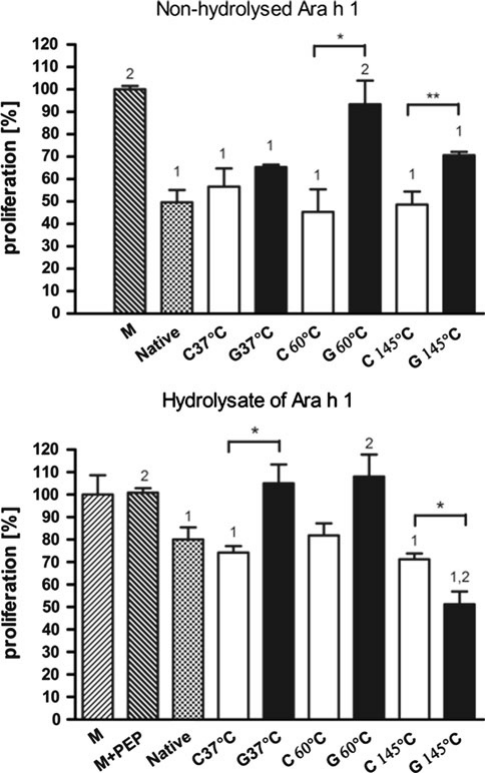
Proliferation of Caco-2 cells incubated with non-hydrolysed and hydrolysed Ara h 1. The percentage of proliferation is presented in the relation to the control proliferation (100 %) of cells incubated in pure medium. Two independent experiments for two different batches of treated Ara h 1 were performed in triplicate. Data were expressed as mean ± SD. Statistical differences between the samples on the level *p < 0.05; *1*—statistical differences between each sample and medium; *2*—statistical differences between each sample and native Ara h 1(N); *M* cells incubated in pure medium; *M* + *PEP* cells incubated in the medium with addition of pepsin; *N* native (non-modified) protein; *C* control sample, Ara h 1 treated without glucose; *G* glycation, Ara h 1 treated with the presence of glucose

First, we determined the optimal conditions for cell-based experiments by testing a range of concentrations of Ara h 1 ranging from 25 to 1000 μg/ml and different times of incubation with Caco-2 cells. The concentration of 500 μg/ml and 1.5-h-long incubation time were considered as optimal (Fig. 4). Incubation of Caco-2 cells in the presence of non-hydrolysed Ara h 1 inhibited their proliferation by 51 %. No temperature/time-dependent differences for the mode of action of Ara h 1 treated without glucose were observed. Treatment of Ara h 1 at 37 °C with glucose did not influence the proliferation of Caco-2 cells. No differences were observed between proliferation rate of cells incubated with Ara h 1 treated in the presence and without glucose. The significant differences were observed in the effect on the Caco-2 proliferation rate between Ara h 1 heated without glucose and glycated at the temperature of 60 and 145 °C (Fig. 4). The proliferation rate of Caco-2 cells incubated with glycated Ara h 1 was comparable to the medium control in the case of treatment at 60 °C.

The addition of hydrolyzate of native Ara h 1 to the culture medium resulted in 20 % decrease of Caco-2 cells proliferation. No temperature/time-dependent differences for the mode of action of Ara h 1 treated without glucose were observed. The incubation of Caco-2 cells with hydrolyzate of Ara h 1 heated without glucose, for all studied time/temperature conditions, as well as Ara h 1 glycated at 145 °C resulted in a significant decrease of proliferation ranging from 18 to 49 % (Fig. 4). The hydrolysate of Ara h 1 glycated at 37 and 60 °C did not exert any effect on Caco-2 proliferation when compared to cells incubated in pure medium. This result was significantly different than the effect observed for Ara h 1 glycated at 145 °C.

#### Secretion of IL-8 by the Caco-2 cells incubated with Ara h 1

**Fig. 5:**
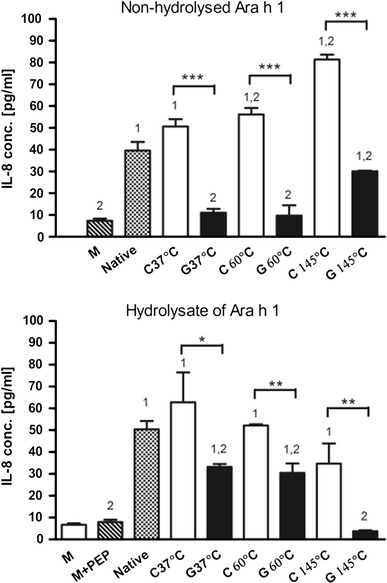
Secretion of IL-8 by human colon carcinoma cell line (Caco-2) incubated with Ara h 1. Secretion of IL-8 by Caco-2 cells incubated with non-hydrolysed and hydrolysed Ara h 1. Two independent experiments for two different batches of treated Ara h 1 were performed in triplicate. Data were expressed as mean ± SD. Statistical differences between the samples on the level *p < 0.05; *1*—statistical differences between each sample and medium; *2*—statistical differences between each sample and native Ara h 1(N); *M* cells incubated in pure medium; *M* + *PEP* cells incubated in the medium with addition of pepsin; *N* native (non-modified) protein; *C* control sample, Ara h 1 treated without glucose; *G* glycation, Ara h 1 treated with the presence of glucose

The level of IL-8 secreted by Caco-2 cells incubated with pure medium and medium with the addition of pepsin solution was estimated to be approximately 6.6 pg/ml and was considered as the control level. In the pre-experiments, we analysed the concentration range for Ara h 1 (25, 100, 250, 500 and 1,000 μg/ml) to choose the optimal one (25 μg/ml). Incubation of Caco-2 cells with native Ara h 1 increased the level of secreted IL-8 which was fivefold higher when compared to the medium control (Fig. 5). The stimulatory properties of Ara h 1 were retained after pepsin hydrolysis (7.5-fold increase of IL-8 secretion when compared to control level). Heating in the absence of glucose did not influence the mode of action of Ara h 1, while MR products, in all tested time/temperature conditions, significantly reduced the secretion of IL-8. Incubation of Caco-2 cells with Ara h 1 glycated at 37 and 60 °C inhibited the secretion of IL-8 by Caco-2 cells and reduced it to the level of the medium control. However, after pepsin hydrolysis, this effect was less pronounced. It is worthwhile to underline that no significant differences in mode of action of Ara h 1 treated at 37 and 60 °C were observed both in the case of non-hydrolysed and hydrolysed protein. The Ara h 1 glycated at the temperature of 145 °C exerted an opposite effect when compared to Ara h 1 glycated at both lower temperatures.

## Discussion

In this study we examined three different conditions of MR on biochemical properties and biological activity of Ara h 1. We demonstrated temperature/time-dependent differences in biochemical characteristics of Ara h 1 glycated at different conditions, which is attributed to different stages of MR and diversity of obtained products. In our previous study on hazelnut 7S globulin, we applied identical temperature/time-treatment conditions as in this study to assess the effect of the MR [15] in terms of its three main phases (early, advanced and final stage) [40, 41]. An effective glycation of Cor a 11 occurred at all investigated conditions [15], fully corroborating observations of this study on Ara h 1. MR at 37 °C did not influence the specific IgG or IgE binding to Cor a 11 [15], suggesting minimal structural changes under these treatment conditions. Analysis of the physicochemical properties of Ara h 1 glycated at 37 °C, such as the lack of brown colour development (Table 1) and no differences between UV–vis spectra of Ara h 1 treated in and without glucose (data not shown), suggest that under these conditions MR did not proceed to the advanced stage. Pedrosa et al. [32], studying the influence of glycation at 37 °C during 50 h on pea 7S globulin, showed that glycation at this mild condition does not significantly change the oligomeric structure of vicilin. However, the glycated vicilin derivatives appeared to be more stable most probably due to covalent attachment of carbohydrate moieties to the protein surface and the participation of carbohydrate hydroxyl groups in intersubunit hydrogen bonding [32]. The results presented by Pedrosa et al. seem to be consistent with data obtained for Ara h 1 glycated at 37 °C; however, the stability of the protein was not directly examined. Fluorescence and browning development in MR are generally used as indicators of the reaction rate and formation of MRPs [34, 42, 43]. We observed both an enhancement of fluorescence and brown colour development in the samples glycated at 60 and 145 °C indicating the advanced and final stage of MR. The low level of fructosamine compared to protein-bound fluorescent compounds (ratio about 3:1) indicate a formation of advanced Maillard reaction products (AGEs) at 60 °C. This is consistent with previous findings showing formation of AGEs during the first 24 h of protein incubation at 60 °C [34]. An increased browning intensity of Ara h 1 glycated at 145 °C correlated with decreased intensity of protein-bound fluorescence, confirming the hypothesis of various authors that fluorescent compounds are precursors for browning products [44, 45]. The kinetics and mechanism of the formation of both colour and fluorescent compounds seem to be highly material- and condition-dependent [43, 46, 47], making speculations about the nature of formed fluorescent and brown compounds very difficult. It has been shown that above 100 °C, the degradation of the Amadori product is faster than its formation [40] what most probably also took place during the glycation of Ara h 1 at 145 °C as suggested by the relations between fructosamine, protein-bound fluorescence and colour development. The diversity and heterogeneity of low-molecular products formed during glycation of Ara h 1 at 145 °C were corroborated by a smear observed on the SDS-PAGE. Similar effects of MR that occurred at temperatures above 100 °C were observed also by other authors [11, 15]. The products obtained through the glycation of Ara h 1 at 60 °C as well as 37 °C seem to be more homogenous as shown on SDS-PAGE. The results obtained due to biochemical analysis indicate temperature/time-dependent differences in the type of obtained MRPs as well as in the protein structure influenced by the temperature. Those results were reflected in biological activity of processed Ara h 1. We showed that high temperature itself (60 and 145 °C) did not alter the DH of Ara h 1 significantly (Fig. 4) while glycation influenced the susceptibility of Ara h 1 to pepsin hydrolysis in a condition-dependent way. The addition of glucose to the system led to more pronounced changes in the Ara h 1 structure caused by the modification of amino acids such as phenylalanine, tyrosine and tryptophan [48–50] that are involved in maintaining tertiary structure of Ara h 1 [8, 48]. The glycation-induced conformational changes of Ara h 1 modified the access of pepsin to its cleavage sites resulting in 41 % decrease of DH in the case of treatment at 60 °C and a reduction of DH to 5 % in the case of glycation at 145 °C. It has been previously shown that advanced stages of MR promote the cross-linking of Ara h 1 which makes the protein progressively more insoluble and unextractable [10, 11, 15, 48]. Enzymatic hydrolysis of cross-linked Ara h 1 most probably generated so-called ‘limit-peptide fragments’ which prevents further hydrolysis because the peptide around the cross-linking site are inaccessible to the hydrolytic enzymes [51]. The literature data on the influence of MR on susceptibility of Ara h 1 to enzymatic hydrolysis are not consistent [10, 11]; however, the studies of Seiquer et al. [52] provide a new point of view on the effects of consumption of MRP-rich diets on dietary nitrogen utilization. Authors showed a negative effect of diet rich in MR products on protein digestibility and pointed particularly at the long-term effects of dietary MRPs on nitrogen utilization [52].

Data from in vitro and in vivo studies suggest that most of AGEs escape digestion in the upper gastrointestinal tract and are mainly recovered in the faeces [53]. Reaching the intestines, glycopeptides may influence the metabolic activity of enterocytes as well as physiological response of intestinal microbiota thus having an impact on the overall health status [17]. We showed a significant anti-proliferative activity of non-hydrolysed Ara h 1 on Caco-2 cells which increased at higher protein concentrations (data not shown). The anti-proliferative potential of native Ara h 1 was significantly reduced by hydrolysis of the protein. This fact may be due to damage to protein sequences that display an anti-proliferative effect. Moreover, the peptides formed during the hydrolysis are a source of nutrients for Caco-2 cells what may additionally reduce the anti-proliferative effect. The anti-proliferative mode of action of peanut 7S globulin seems to be consistent with data obtained by Jia et al. [54] who demonstrated a decreased proliferation rate of intestinal epithelial cells isolated from 28-day-old piglets as the result of incubation with soy 7S globulin. Caco-2 proliferation rate expressed by the cell viability (direct cell counting, measurement of total cell protein or DNA) or its enzymatic activity (MTT and WST-1 tests) often forms the basis for an assessment of cytotoxic potential of examined substances [14, 22, 28, 29, 55]. Gerlier and Thomasset observed that the increase in mitochondrial activity was independent of the increase in DNA synthesis [56], which proves that the proliferative events can be independent from mitochondrial activity thus complicating comparison of different studies. Some authors imply additional methods, for example cell membrane damage tests: crystal violet assay and lactate dehydrogenase (LDH) leakage [27] leading to a more accurate assessment of cell physiological condition. However, these classical methods measure both cell viability and membrane permeabilization but do not quantify the proportion of apoptotic target cells compared to normal vital cells and dead cells and are not able to distinguish early apoptotic cells from highly apoptotic and necrotic ones [57, 58]. More precise and consistent methods for assessment of food compounds cytotoxicity are needed to enable the comparison of results presented by different research groups. Therefore, based on anti-proliferative effects of native Ara h 1, we can speculate that Ara h 1 possess cytotoxic properties against intestinal epithelia cells. Our results indicate that protein-bound MRPs obtained during glycation of Ara h 1 at 60 and 145 °C may attenuate the anti-proliferative effect of native Ara h 1. To clarify this hypothesis, independently of BrdU test, more precise cytometric methods should be applied [59, 60].

We demonstrated a stimulation of IL-8 production by Caco-2 cells incubated in the presence of native Ara h 1. These results seem to be consistent with an effect of Ara h 1 on the proliferation of Caco-2 cells. An increase of IL-8 secretion by Caco-2 cells may be a cellular response to compensate for anti-proliferative effects of this protein. Those findings confirm the ability of Ara h 1 to induct a local inflammatory response in gut mucosa what may be related to the high allergenic potential of this molecule. The stimulatory properties of the native form of Ara h 1 were retained after pepsin hydrolysis which suggests that the fragments responsible for this effect were protected from digestion. MRPs formed during glycation of Ara h 1, at all studied time/temperature conditions (37, 60, 145 °C), acted as an inhibitor of IL-8 secretion by Caco-2 cells. Moreover, the results observed for Ara h 1 glycated at 37 and 60 °C demonstrate a consistent effect as no differences between the mode of action of those samples were observed. However, we demonstrated different stage of MR for each tested condition what was also confirmed on SDS-PAGE. Therefore, inhibition of IL-8 secretion observed as the result of incubation of Caco-2 cells with Ara h 1 glycated under all three tested conditions may be caused by different substances from a chemical point of view. However, in the light of obtained results, a more detailed chemical analysis of the products of glycation of Ara h 1 together with the determination of their anti-inflammatory effect is needed. Our results suggest that an inhibitory effect is more prominent before pepsin digestion than after. So most probably, the bigger structures play a crucial role in IL-8 inhibition. However, the glycopeptides obtained after pepsin hydrolysis did not lose their biological properties responsible for IL-8 inhibition. Our findings are consistent with the previous results which showed that low-molecular MR fractions may reduce the inflammation in Caco-2 cells induced with IFN-γ + PMA by the transcriptional down-regulation of genes involved in the NF-κB pathway as well as the translational inhibition of iNOS and IL-8 expression [21, 22]. Other studies showed that transcription factor NF-κB which plays a critical role in inflammation and cancer development [61] may be activated via the receptor for advanced glycation end products (RAGE) [62]. Therefore, the advanced MRPs, formed due to glycation of Ara h 1, may exert their effect on Caco-2 through RAGE which is indeed expressed by Caco-2 cells [23]. As it was shown for protein-linked CML (*N*-carboxymethyl-lysine), MRPs may induce the activation state of p44/42 (ERK1/2) mitogen-activated protein kinases (MAPKs) in Caco-2 cells via interaction with RAGE [23]. MAPKs were shown to regulate cellular proliferation in Caco-2 cells [63] making it possible that AGEs obtained during glycation of Ara h 1 stimulated intestinal epithelial cell proliferation via MAPK activation. However, this speculation requires further studies. Some authors showed an anti-proliferative action of MRPs [22, 50, 64], while Jing and Kitts [14] showed no significant toxicity of casein glycated at 55 °C during 28 days to Caco-2 cells. These findings suggest that the way of action of MRPs on the proliferation of Caco-2 cells depends on the biochemical structure of a protein and the type of obtained products.

We showed that glycation of Ara h 1 at high temperatures (60 and 145 °C) led to advanced stages of MR which decreases the degree of protein hydrolysis and modulates the interaction of Ara h 1 with Caco-2 cells. However, glycation of Ara h 1 at 37 °C, which was shown to cause least biochemical changes, also modulated the interaction of modified Ara h 1 with gut epithelium by inhibition of IL-8 secretion. It is important to note that conclusions concerning the bioactive properties of MRPs, susceptibility to pepsin hydrolysis and interaction with gut epithelium underestimate the importance of the temperature/time conditions of MR. Our studies showed that the MRPs of Ara h 1 obtained in all three studied time/temperature conditions may influence the “pro-inflammatory network” in the human intestinal mucosa, creating the need of further studies to determine the chemical structure of these products, their anti-inflammatory mechanisms of action as well as interaction with gut microbiota.
